# 1D Pre‐Acquisition Navigator Correcting Respiratory‐Induced Field Fluctuations in Multi‐Echo Gradient‐Echo Imaging of the Thoracic Spinal Cord

**DOI:** 10.1002/mrm.70528

**Published:** 2026-07-24

**Authors:** Alicia E. Cronin, Alexandre D'Astous, Nathan Williams, Antoine Guénette, Aimee Salakhov, Seth Stubblefield, Colin D. McKnight, Lipika Narisetti, Subramaniam Sriram, Seth A. Smith, Ryan K. Robison, Guillaume Gilbert, Julien Cohen‐Adad, Kristin P. O'Grady

**Affiliations:** ^1^ Vanderbilt University Institute of Imaging Science, Vanderbilt University Medical Center Nashville Tennessee USA; ^2^ Department of Radiology and Radiological Sciences Vanderbilt University Medical Center Nashville Tennessee USA; ^3^ NeuroPoly Lab Institute of Biomedical Engineering, Polytechnique Montréal, Quebec Canada; ^4^ Centre de Recherche du CHU Sainte‐Justine Université de Montréal Montréal Quebec Canada; ^5^ Department of Biomedical Engineering Vanderbilt University Nashville Tennessee USA; ^6^ Neuroimmunology Division, Department of Neurology Vanderbilt University Medical Center Nashville Tennessee USA; ^7^ Philips Cambridge Massachusetts USA; ^8^ MR Clinical Science Philips Healthcare Canada Mississauga Ontario Canada; ^9^ Functional Neuroimaging Unit, CRIUGM Université de Montréal Montréal Quebec Canada; ^10^ Mila–Quebec AI Institute Montréal Quebec Canada

**Keywords:** artifacts, B_0_ field fluctuations, multi‐echo gradient‐echo, navigator correction, spinal cord

## Abstract

**Purpose:**

In the spinal cord (SC), multi‐echo gradient echo (ME‐GRE) increases gray (GM) and white matter (WM) contrast and improves sensitivity to lesions in people with multiple sclerosis (pwMS). However, SC ME‐GRE is susceptible to breathing‐induced field fluctuations, causing ghosting artifacts and signal loss. Recent work introduced a 1D phase navigator following the last echo to measure field variations; however, susceptibility to phase wrapping increases at longer echo times. We propose a 1D phase navigator preceding the first echo, reducing phase accumulation and eliminating the need for respiratory monitoring.

**Methods:**

ME‐GRE data covering the lower (T9–T12 vertebrae) and upper (T4–T8 vertebrae) thoracic SC were acquired in 20 healthy volunteers and 3 pwMS at 3T. Standard and navigator‐corrected images were acquired in the same acquisition. To evaluate image quality, WM and GM signal‐to‐noise ratio (SNR), WM/GM contrast‐to‐noise ratio (CNR), and background ghosting signals were measured and compared between the two reconstructions. Both were blindly assessed for artifacts, structural delineation, and diagnostic confidence in pwMS.

**Results:**

Navigator correction significantly increased GM and WM SNR and CNR, reduced posterior ghosting across both thoracic regions, and significantly reduced artifacts while increasing structural delineation. Preliminary evaluation in three pwMS showed consistent improvements in artifact mitigation, structural delineation, and lesion conspicuity with navigator correction, providing proof‐of‐concept for potential clinical application.

**Conclusion:**

A 1D navigator prior to the first echo reduces ghosting and improves thoracic SC image quality without respiratory monitoring. This approach could improve the diagnostic value and enhance the reliability of thoracic SC ME‐GRE.

## 1 Introduction

T2*‐weighted multi‐echo gradient echo (ME‐GRE) sequences are commonly used for spinal cord imaging whereby multiple gradient echoes are acquired at various echo times and averaged to increase contrast between gray matter (GM) and white matter (WM) [1, 2]. It has also been shown to have greater sensitivity to lesion detection in multiple sclerosis (MS) [3, 4] and reduced cerebrospinal fluid (CSF) flow‐related artifacts [5] compared to the conventional T2‐weighted turbo spin‐echo (TSE) acquisition. However, challenges of ME‐GRE in the spinal cord include relatively longer acquisition times compared to conventional T2‐weighted TSE acquisitions and greater sensitivity to B_0_ field variations due to magnetic susceptibility differences between tissue and air [6], which cause motion‐ and respiration‐induced artifacts and signal loss. In the cervical spinal cord, B_0_ field shifts from respiration can be as significant as 74 Hz at 3T at the C7 level [7] and are temporally varying. These field shifts can cause shot‐to‐shot phase discrepancies, resulting in signal loss and motion‐induced ghosting artifacts.

Several approaches have been established to address these breathing‐induced B_0_ fluctuations; however, most have been performed in the brain [8, 9, 10, 11, 12, 13, 14, 15]. Fewer studies have been performed in the spinal cord. In the cord, previous studies have explored correcting B_0_ field shifts using respiratory bellows data [16, 17, 18], and also real‐time and dynamic shimming methods [16, 17, 19]. However, specialized hardware is required for these more advanced shimming techniques, which is unavailable at most sites, and the application of these various methods has been limited, even with the potential image quality gain. A recent ME‐GRE study performed by Beghini et al. [20] investigated the use of a 1D phase navigator acquired after the last echo in each repetition time (TR) to capture the central *k*‐space line, allowing for field variation measurements across time. However, because the 1D navigator was incorporated after the last echo in each TR, the measured phase reflects both dynamic fluctuations and accumulated TE‐dependent phase evolution, which, at these extended echo times, may require unwrapping. This necessitates the use of external respiratory bellows to track the breathing cycle to correct for these phase wraps [20], adding hardware requirements and additional data processing that limits clinical adoption.

In this study, we propose a navigator processing pipeline in which the 1D phase navigator is placed prior to the first echo [21], thereby reducing phase accumulation due to the shorter echo time (TE) at the time of the applied phase navigator and eliminating the need for external respiratory monitoring. We assessed the impact of this proposed navigator‐correction reconstruction on ME‐GRE imaging of the thoracic spinal cord at 3T. The thoracic spinal cord was the acquisition region of interest due to the pronounced susceptibility differences and large, temporal B_0_ field variations. These challenges have been previously demonstrated by Beghini et al. [20] who reported the largest image artifacts in the C7‐T1 and T8–T11 regions, where the spinal cord borders the lungs. Because of these challenges, the thoracic region remains relatively understudied, and improving the robustness to respiration‐induced field fluctuations in the thoracic spinal cord will have both technical and clinical relevance.

## 2 Methods

### 2.1 Data Acquisition

All methods were carried out in accordance with the relevant guidelines and regulations, and the Vanderbilt University Institutional Review Board approved experimental protocols. Prior to imaging, written informed consent was acquired. Twenty healthy volunteers (13 females; mean age ± SD: 30.4 ± 8.1 years; mean BMI ± SD: 25.1 ± 5.2 kg/m^2^) participated in this study, along with three persons with relapsing–remitting MS (pwRRMS) (3 females; mean age ± SD: 45.3 ± 5.5 years; mean BMI ± SD: 23.8 ± 4.2 kg/m^2^).

Scanning was performed on a 3T whole‐body Philips dStream Achieva MR scanner (Philips, Best, Netherlands), with a 2‐channel body coil for signal transmission and an integrated 12‐channel posterior spinal cord array for reception. A sagittal T2‐weighted TSE sequence was acquired for field‐of‐view (FOV) positioning of the ME‐GRE acquisitions (sensitivity encoding (SENSE) = 1.4, TR/TE = 2765/120 ms, resolution = 1.0 × 1.1 × 5 mm^3^, number of slices = 11, FOV = 270 × 270 mm^2^, and NSA = 2). A single‐echo GRE navigator was inserted before the first echo (navigator TE = 5.15 ms) of the axial ME‐GRE sequence to measure instantaneous B_0_ fluctuations (Figure 1A), with the following parameters: compressed‐SENSE (CS) = 2, FOV = 180 × 180 mm^2^, resolution = 0.65 × 0.65 × 4 mm^3^, number of slices = 25, TR = 485 ms, TE = {11, 18.1, 25.2} ms, 3 echoes, FA = 43°, NSA = 3, scan time = 8 min 36 s. To confirm that the navigator was capturing respiratory‐induced B_0_ fluctuations rather than noise, an independent validation experiment was performed in one participant (female, 35 years old). A dynamic single‐slice sagittal Dixon GRE scan was acquired with the following parameters: FOV = 250 × 250 mm, resolution = 3 × 3 × 8 mm, TE = {1.35, 2.35} ms, Δ*t* = 1.0 s. Dynamic B_0_ maps were computed from the resulting phase images, and regional B_0_ time courses were extracted from ROIs placed at vertebral levels T9, T11–T12, and L1. A concurrent respiratory bellows trace was acquired simultaneously.

**FIGURE 1:**
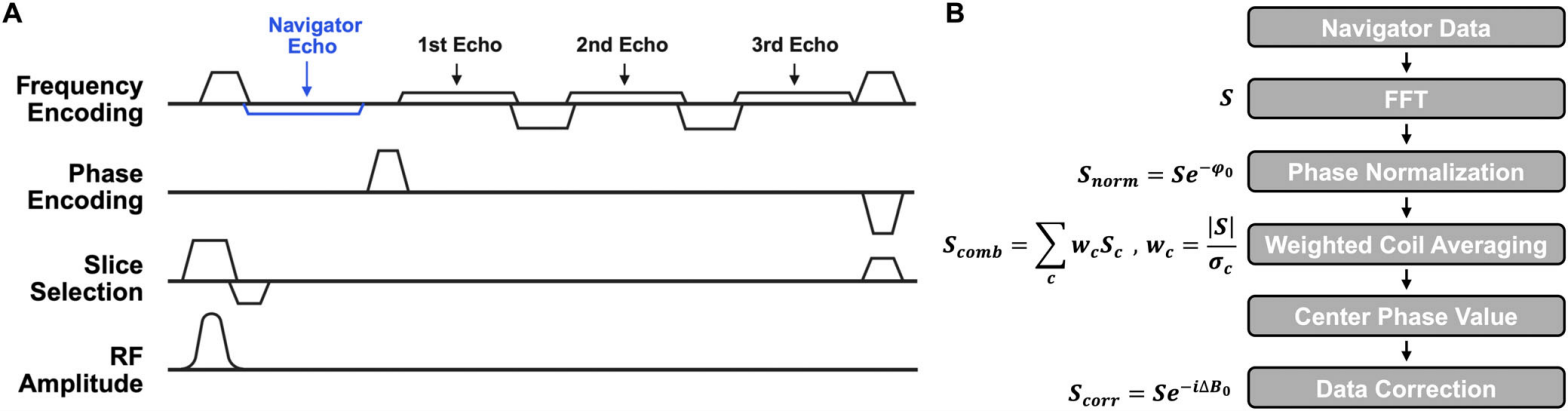
(A) Pulse sequence diagram illustrating the ME‐GRE sequence with the navigator incorporated before the first echo of each echo series. (B) ME‐GRE pipeline of navigator‐based correction in the spinal cord. Raw data was converted to image space using a 1D fast Fourier transform. Static phase contributions were removed, and dynamic B_0_ fluctuations were isolated by phase normalization. Within each slice, an SNR‐weighted averaging across coils was computed. Finally, using the center phase value of the combined navigator signal, the expected phase accrual induced by the estimated phase field‐estimates was removed by demodulation, producing the navigator‐reconstructed image.

In all 20 healthy volunteers, multi‐slice navigated ME‐GRE were acquired in the lower portion of the thoracic spinal cord (spanning vertebral levels T9–T12), while in 10 healthy volunteers, navigated ME‐GRE was also performed in the upper portion of the thoracic spinal cord (spanning vertebral levels T4–T8). In the three pwRRMS, all participants had both image FOV acquisitions performed. Each FOV was angled to maximize the number of slices perpendicular to the cord, thereby minimizing partial‐volume effects. Figure 2A demonstrates an example of the FOV positioning for one healthy volunteer.

**FIGURE 2:**
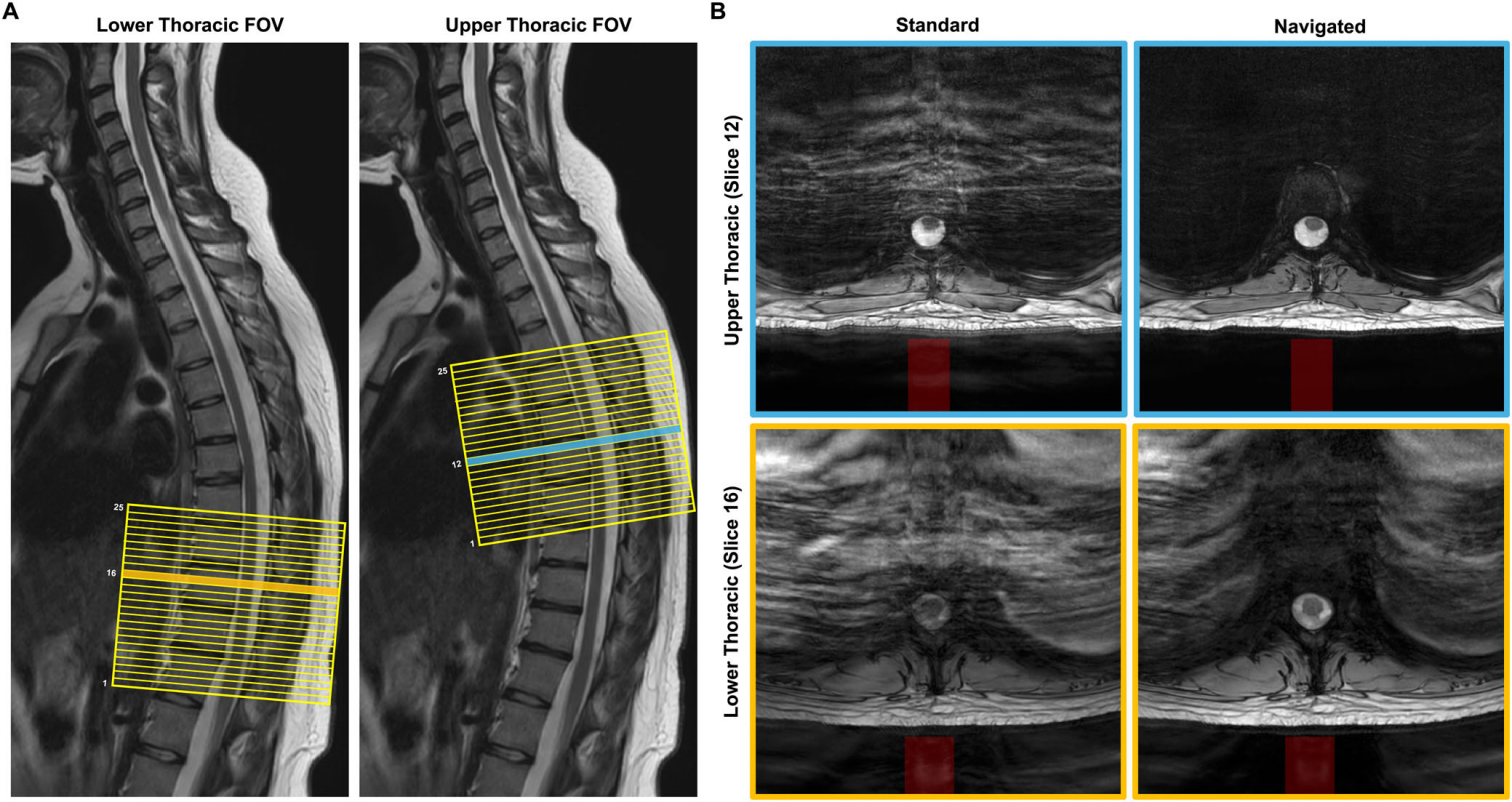
(A) Representative T2‐weighted sagittal image of one healthy participant (28‐year‐old female), with axial FOV overlaid in yellow, and highlighted example slices in orange and blue, in the lower and upper thoracic spinal cord, respectively. (B) Corresponding highlighted slices in the upper (blue) and lower (orange) thoracic spinal cord with both the standard‐ and navigator‐corrected reconstructed images. The red ROI is the rectangular posterior background mask used to measure ghosting signal intensity, identified by Otsu thresholding of the posterior tissue boundary.

### 2.2 Navigator Processing

An overview of the navigator processing pipeline is shown in Figure 1B, modified from work performed by Beghini et al. [20], and each step is performed independently for each slice. A 1D fast Fourier transform (FFT) was applied to each navigator readout to convert the data to image space [10]. Because the FOV is centered around the spinal cord, only the center‐pixel of the FFT output was used to estimate the global slice‐wise field fluctuation in the spinal cord.

To remove static phase contributions (static B_0_, B_1_^+/−^) and isolate the dynamic B_0_ fluctuations, normalization of each navigator line to the first acquired navigator line in that slice was performed:

(1) $$S_{\mathrm{norm}}=\text{Sexp}\left[-\varphi_{0}\right]$$

where $\varphi_{0}$ is the phase of the reference navigator line, S is the FFT navigator data, and $S_{\mathrm{norm}}$ is the data normalized.

Within each slice, there is substantial variability in the signal contributions from different receiver coils. To maximize the SNR of the navigator data, an SNR‐weighted averaging across the coils was computed:

(2) $$S_{\text{comb}}=\sum_{c}w_{c}S_{c}$$

where $S_{c}$ is the center‐pixel navigator signal from coil *c* and the coil weights, $w_{c}$, were computed as:

(3) $$w_{c}=\frac{\left|S\right|}{\sigma_{c}}$$

with $\sigma_{c}$ is the standard deviation of the noise acquisition for each coil.

The phase of the combined navigator signal,

(4) $$\emptyset_{\mathrm{nav}}=\arg\left(S_{\text{comb}}\right)$$

was converted to a slice‐wise phase field‐estimate scaled in Hz:

(5) $$\Delta B_{0}=\frac{\emptyset_{\mathrm{nav}}}{2\pi {\mathrm{TE}}_{\mathrm{nav}}}$$

Because the navigator was acquired before the first echo and at short TE (5.15 ms), the assumption was made that $\emptyset_{\mathrm{nav}}$ did not require temporal unwrapping. For each readout sample occurring at time *t* relative to the start of the first echo, the expected phase accrual induced by the estimated phase field‐estimates was removed by demodulation [22]:

(6) $$S_{\text{corr}}\left(t\right)=S_{\text{uncorr}}\left(t\right)\exp\left[-i\Delta B_{0}t\right]$$

This correction was applied to all echoes prior to image reconstruction. By acquiring the navigator readout at a short TE, the estimate of the global frequency offset is obtained before substantial spatially varying phase dispersion develops, eliminating the need for external respiratory monitoring or triggering. Images were reconstructed in real‐time on the scanner without (standard) and with (navigated) correction.

### 2.3 Quantitative Analysis

Multiple image quality metrics were computed to evaluate the performance of the navigator correction. Using Spinal Cord Toolbox v7.0 [23], spinal cord and GM masks were created on the navigator‐corrected image using *sct_deepseg* and manually checked. WM masks were created by subtracting the GM mask from the whole cord mask. Signal‐to‐noise ratio (SNR) was calculated for both the WM and GM using the following equation:

(7) $$\mathrm{SNR}=\frac{\text{mean}\left(S\right)}{\mathrm{std}\left(S\right)}$$

where *S* is the signal within the tissue of interest. The contrast‐to‐noise ratio (CNR) was calculated between GM and WM:

(8) $$\mathrm{CNR}=\frac{\left|\text{mean}\left(S_{\mathrm{WM}}\right)-\text{mean}\left(S_{\mathrm{GM}}\right)\right|}{\sqrt{\mathrm{std}{\left(S_{\mathrm{WM}}\right)}^{2}+\mathrm{std}{\left(S_{\mathrm{GM}}\right)}^{2}}}$$

For both SNR and CNR, a single measurement was acquired for the whole image and both the mean and median values were obtained.

Ghosting was quantified by measuring residual signal in a posterior background region defined automatically from navigated images. Specifically, using the spinal cord segmentations, the posterior tissue boundary was identified using Otsu thresholding. For each slice, a rectangular background mask, centered on the cord and of 1 cm width in the right–left (R‐L) direction and extending posteriorly to the edge of the field of view was generated, where an example is shown in Figure 2B. Mean and max signal intensity within this mask were used as ghosting metrics.

To assess how centered the spinal cord was in the readout direction (i.e., R‐L direction), the centroid of the segmentation mask was computed for each axial slice. The signed displacement between the mask centroid and the image FOV center was calculated in the R‐L direction for each slice as the centroid position minus the image center, in voxels. The width of the spinal cord mask in the R‐L direction was also computed per slice as the number of voxels spanning the R‐L width to contextualize displacement magnitude relative to the spinal cord size. Note that all slices were included in all ROI analyses.

### 2.4 Qualitative Analysis

Finally, to further assess image quality, two neuroradiologists (McKnight and Stubblefield) blindly qualitatively rated all image volumes based on the 5‐point Likert Scale. For healthy volunteers, images were assessed on both artifacts and structural visibility. Artifact scoring focused on ghosting in the median plane of each slice (R–L centered), with scores ranging from 1 (poor, severely degraded) to 5 (excellent, no visible artifacts). Structural assessment proceeded via qualitative visualization of the spinal cord and apparent GM to WM contrast, with scores ranging from 1 (poor visibility) to 5 (excellent visibility). For pwRRMS, an additional qualitative assessment of diagnostic confidence for the image volume was performed, reflecting the reviewer's confidence in the presence or absence of lesions (1 = poor confidence, 5 = excellent confidence).

### 2.5 Pre‐Echo and Post‐Echo Navigator Comparison

To provide a comparison with the previously published navigator‐based approach by Beghini et al. [20], we implemented both the pre‐echo navigator and post‐echo navigator (Beghini et al.) methods in a sub‐analysis using data from one representative healthy participant (female, 35 years old) in the lower thoracic spinal cord. The post‐echo navigator reconstruction incorporated phase unwrapping to ensure a fairer comparison. For each method, standard and navigator‐corrected quantitative image quality metrics (WM SNR, GM SNR, and CNR) were calculated.

### 2.6 Statistics

SNR in the WM and GM, CNR, and ghosting were compared between the standard and navigator‐corrected reconstructions across three groupings: lower thoracic, upper thoracic, and combined thoracic (both the upper and lower thoracic). For the lower and upper thoracic regions, differences between the standard and navigator‐corrected reconstructions were assessed using paired *t*‐tests. For the combined thoracic region, subjects had either one (lower only) or two (lower and upper) measurements for each metric. A linear mixed‐effects model was used to evaluate the differences between the reconstruction methods. The model included reconstruction method as a fixed effect and a subject‐specific random intercept to account for repeated measures. *p* values were adjusted for multiple comparisons across the three anatomical regions using the Benjamini–Hochberg false discovery rate (FDR).

For the blind qualitative assessment, for each assessment category, the two ratings per image were averaged to yield a single score per subject and region. Differences between the standard and navigator‐corrected reconstructions were assessed in the healthy volunteers with a paired *t*‐test for the lower and upper thoracic regions and with a linear mixed‐effects model for the combined region. The linear mixed‐effects model included reconstruction as a fixed effect and a subject‐specific random intercept to account for repeated measurements across regions. *p* values were adjusted using FDR for multiple region comparisons. To evaluate the relation between quantitative image quality metrics (CNR, SNR in WM and GM, and ghosting) and the qualitative assessment, associations between the average Likert scores and the mean quantitative metric were assessed using Spearman rank correlation coefficients in both the upper and lower thoracic spinal cord. Analyses were performed separately for the standard and navigator‐correction reconstructions. To determine whether spinal cord centering accuracy influenced navigator performance, Spearman correlations were computed between the absolute mean displacement in the R‐L direction and four image‐quality metrics (CNR difference, GM SNR difference, WM SNR difference, and ghosting difference, where difference = navigated metric − standard metric). Because of the low sample size of pwRRMS, differences between the standard and navigator‐corrected reconstructions were not statistically compared. All statistics were performed in R version 4.5.1 (R Foundation for Statistical Computing, Vienna, Austria).

## 3 Results

### 3.1 Spinal Cord Centering and Navigator Robustness

Analysis of spinal cord position relative to the FOV center in the R‐L direction revealed variable centering of the cord across slices and participants. In the lower thoracic spinal cord, the absolute mean displacement of the spinal cord mask centroid from the center of the FOV was 7.84 ± 6.82 voxels (median displacement of 6.3 voxels), while the mean displacement in the upper thoracic spinal cord was 8.79 ± 8.22 voxels (median displacement of 6.9 voxels). Figure S1 shows the slice‐wise frequency of displacement in the R‐L direction for both regions, with distributions centered near zero but with spread, including a small number of slices with larger displacement. Given the mean spinal cord width of 31 voxels, the average displacement corresponded to a small fraction of the spinal cord width in both regions. Across both thoracic regions, no significant correlations were found between absolute mean displacement in the R‐L direction and any image‐quality metric (Figure S2), indicating that navigator correction performance was robust to cord positioning variability within the FOV.

### 3.2 Quantitative Image Quality Evaluation

The time dependence of B_0_ at three different vertebral levels is shown in Figure 3. Variations of up to 40 Hz were observed and align with the trace from the respiratory bellows. In all participants, navigator‐correction reconstruction significantly increased both mean GM and WM SNR across all thoracic regions, as demonstrated in Figure 3. For GM, the combined thoracic SNR increased from 12.51 ± 2.96 with standard reconstruction to 17.35 ± 2.27 with navigation (*p* < 0.0001). Similar improvements were observed in the lower thoracic spinal cord (11.34 ± 2.93 vs. 15.56 ± 2.47, *p* < 0.0001) and upper thoracic spinal cord (14.37 ± 4.08 vs. 20.29 ± 4.18, *p* < 0.0001). Similarly, for WM SNR, there was an overall significant increase in SNR in the navigator‐correction reconstruction compared to the standard reconstruction. Specifically, navigation increased SNR in the combined thoracic region from 8.18 ± 2.15 to 10.36 ± 2.05 (*p* < 0.0001), the lower thoracic spinal cord from 7.72 ± 2.20 to 9.76 ± 2.24 (*p* < 0.0001), and in the upper thoracic spinal cord from 8.42 ± 2.34 to 10.69 ± 2.31 (*p* < 0.001). Median SNR values similarly significantly increased in the navigator‐correction reconstruction compared to the standard reconstruction, indicating that improvements were consistent across participants and not driven by outliers.

**FIGURE 3:**
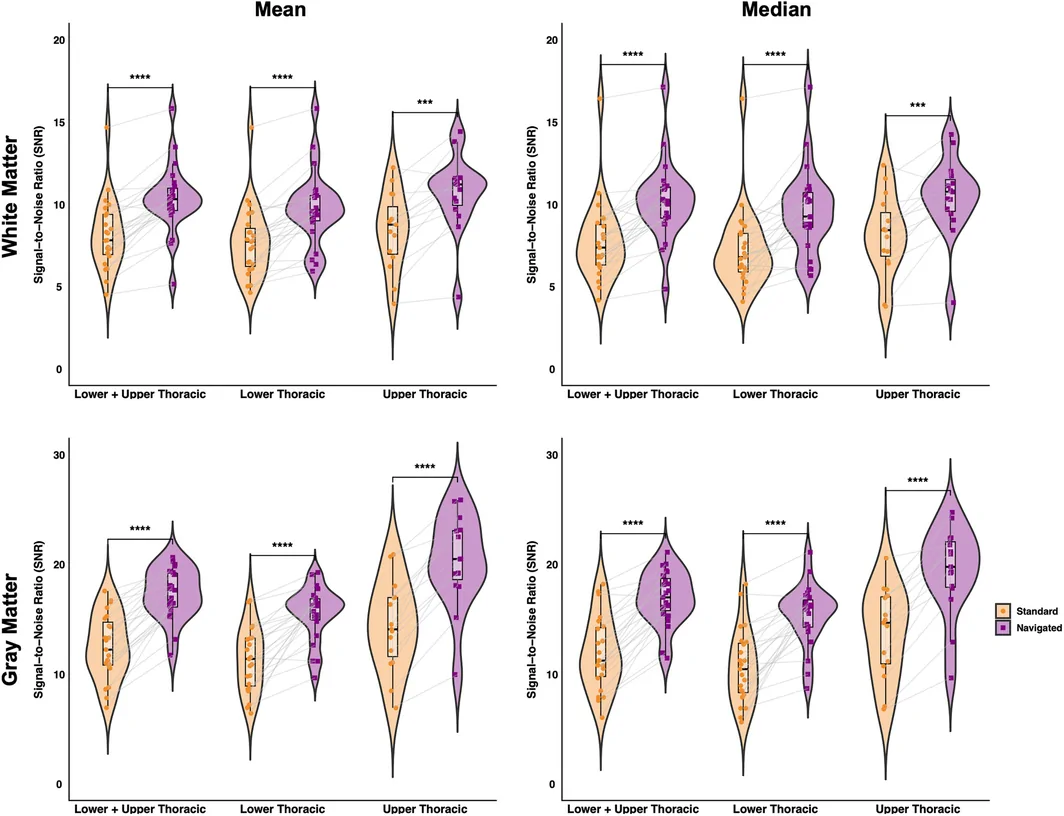
Mean (left) and median (right) signal‐to‐noise ratio (SNR) metrics in the white matter (WM) (top) and gray matter (GM) (bottom) throughout the thoracic spinal cord in both the standard‐ and navigator‐reconstructed images in all participants. In the upper thoracic (*n* = 13), lower thoracic (*n* = 23), and combined region (*n* = 23), the navigator‐reconstructed SNR is significantly greater than the standard SNR. Violin plots show the distribution of the data, with the interquartile range and the median represented by the line. ****p < 0.0001, ***p < 0.001.

CNR between GM and WM also significantly increased across all thoracic regions in the navigator‐correction reconstruction compared to standard reconstruction (Figure 4A). Specifically, in the combined region, the navigator‐correction increased mean CNR to 1.01 ± 0.26 compared to the standard CNR of 0.67 ± 0.18 (*p* < 0.0001). Similarly, mean CNR was higher in the navigator‐correction compared to the standard reconstruction in the lower region (1.00 ± 0.29 vs. 0.65 ± 0.19, *p* < 0.0001) and upper region (0.87 ± 0.20 vs. 0.63 ± 0.15, *p* < 0.001). Median CNR values had similar significant increases in the navigator‐correction reconstruction.

**FIGURE 4:**
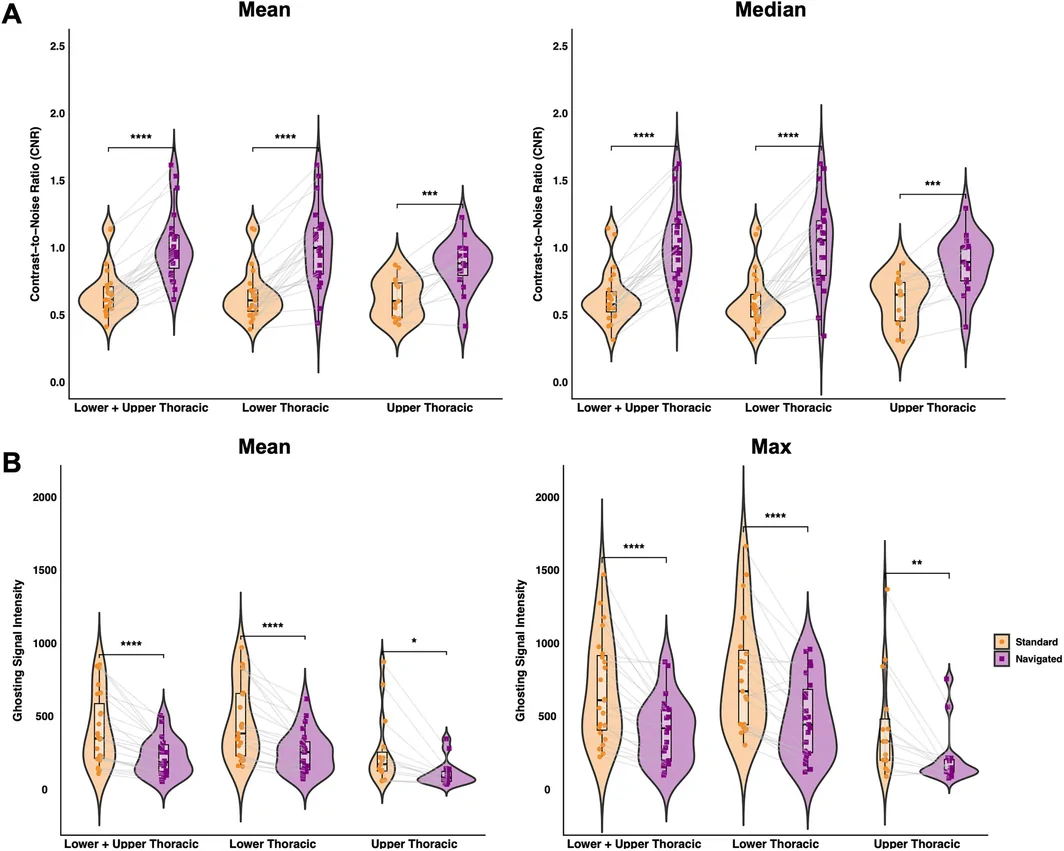
(A) Mean (left) and median (right) contrast‐to‐noise ratio (CNR) metric between white matter and gray matter throughout the thoracic spinal cord in both the standard‐ and navigator‐reconstructed images in all participants. In the upper thoracic (*n* = 13), lower thoracic (*n* = 23), and combined region (*n* = 23), the navigator‐reconstructed CNR is significantly greater than the standard CNR. (B) Mean (left) and max (right) ghosting signal intensity in the posterior region throughout the thoracic spinal cord in both the standard‐ and navigator‐reconstructed images in all participants. In the upper thoracic (*n* = 13), lower thoracic (*n* = 23), and combined region (*n* = 23), the ghosting was significantly reduced in the navigator‐reconstructed image compared to the standard‐reconstructed image. Violin plots show the distribution of the data, with the interquartile range and the median represented by the line. ****p < 0.0001, ***p < 0.001, **p < 0.01, *p < 0.05.

Ghosting in the posterior background region was significantly reduced in the navigator‐correction compared to the standard reconstruction (Figure 4B). In the combined upper and lower thoracic region, there was a significant reduction in the mean ghosting signal in the navigated images compared to standard images (216 ± 125 vs. 405 ± 246, *p* < 0.0001). Mean ghosting signal was also reduced in the navigated images for the lower thoracic spinal cord (257 ± 145 vs. 459 ± 261, *p* < 0.0001) and upper thoracic spinal cord (109 ± 89 vs. 256 ± 243, *p* < 0.05). Similarly, the maximum signal in the posterior ghosting ROI was significantly reduced in the navigator‐correction reconstruction compared to standard reconstruction.

### 3.3 Healthy Volunteers Qualitative Image Quality Evaluation

Figures 5 and S4 demonstrate that the navigator‐correction reconstruction visually reduces the ghosting artifacts in both the upper and lower thoracic regions, as well as provides more uniform signal within tissue, higher contrast between tissues, brighter CSF, and clearer anatomical details. Differences between the standard and navigator‐corrected reconstruction images show that more signal is retained within the spinal cord and CSF when reconstructing using the navigator, as evidenced by the brighter signal in the pairwise difference maps.

**FIGURE 5:**
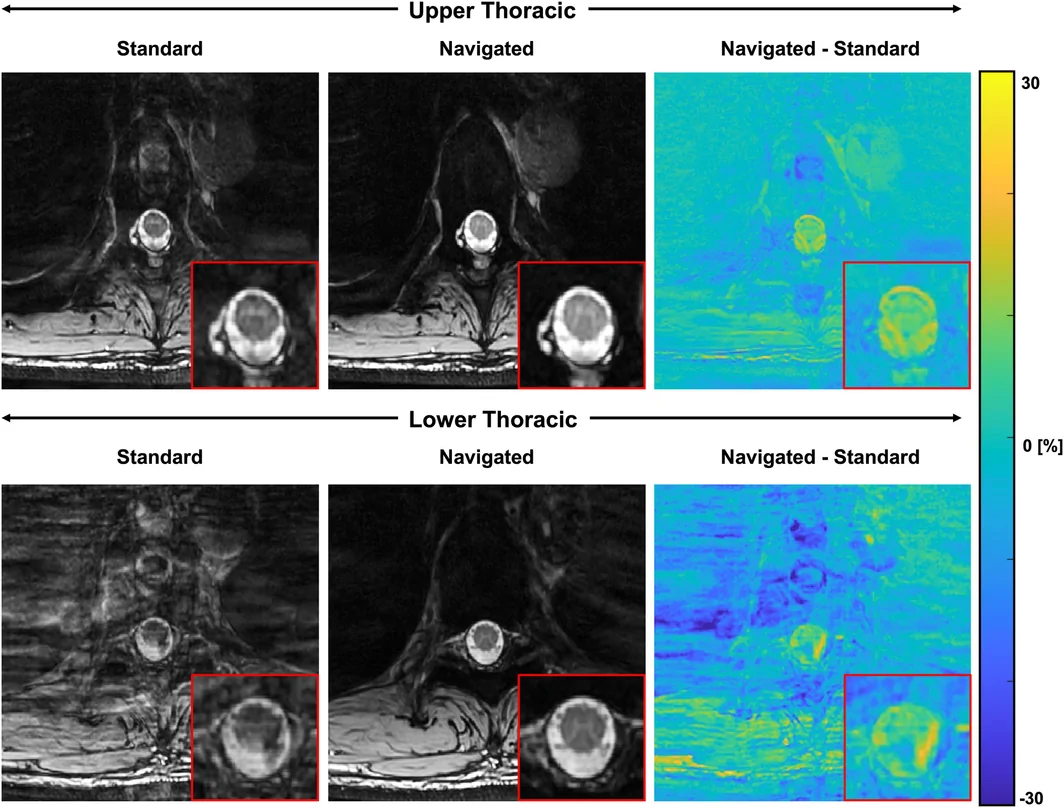
Representative ME‐GRE images from one healthy participant (52‐year‐old male) in the upper and lower thoracic spinal cord, with the standard‐reconstruction (no navigator) and navigator‐corrected reconstruction displayed. Pairwise differences between the standard and navigated reconstructed images were computed after normalization (dividing each image by the maximum intensity value in that image). See Figure S4 for more example images of healthy participants.

In healthy volunteers, a blind qualitative assessment using a Likert scale was performed and demonstrated significant improvement in image quality with navigator‐correction reconstruction compared with standard reconstruction, particularly in artifact reduction. Specifically, in the lower thoracic region, navigated images demonstrated improved artifact reduction (2.33 ± 1.11 vs. 1.80 ± 0.69, *p* < 0.001) and structural delineation scores (2.70 ± 1.11 vs. 1.98 ± 0.80, *p* < 0.001) compared to the standard reconstruction images. In the upper thoracic spinal cord, artifact scores were similarly improved with navigation (3.20 ± 0.95 vs. 2.45 ± 1.05, *p* < 0.001), while structural scores did not differ significantly between reconstruction methods (2.35 ± 0.99 vs. 2.00 ± 0.73, *p* = 0.29). When combined across both the upper and lower thoracic regions, navigator‐corrected reconstruction produced higher artifact reduction (2.62 ± 0.95 vs. 2.02 ± 0.87, *p* < 0.0001) and structural delineation scores (2.58 ± 1.08 vs. 1.98 ± 0.77, *p* < 0.001). Table 1 summarizes these results.

**TABLE 1:** Mean (±SD) Likert scores of the two scorers in the healthy volunteers (*n* = 20) for both the standard‐ and navigator‐corrected reconstructions in the lower, upper, and combined thoracic regions. For each Likert score, the percentage of participants who improved, worsened, or remained the same after navigator‐corrected reconstruction is presented.

		Standard mean ± SD [min, max]	Navigated mean ± SD [min, max]	*p*	% Improved	% Worsened	% Same
Lower thoracic	Artifact	1.80 ± 0.69 [1, 3]	2.33 ± 0.83 [1, 3.5]	^***^	75% (15/20)	5% (1/20)	20% (4/20)
	Structural	1.98 ± 0.80 [1, 3.5]	2.70 ± 1.11 [1, 5]	^***^	65% (13/20)	10% (2/20)	25% (5/20)
Upper thoracic	Artifact	2.45 ± 1.05 [1, 3.5]	3.20 ± 0.95 [1, 4]	^***^	90% (9/10)	0% (0/10)	10% (1/10)
	Structural	2.00 ± 0.73 [1, 3]	2.35 ± 0.99 [1, 4]	NS	40% (4/10)	20% (2/10)	40% (4/10)
Lower + upper thoracic	Artifact	2.02 ± 0.87 [1, 3.5]	2.62 ± 0.95 [1, 4]	^****^	80% (24/30)	3.3% (1/30)	16.7% (5/30)
	Structural	1.98 ± 0.77 [1, 3.5]	2.58 ± 1.08 [1, 5]	^***^	57% (17/30)	13% (4/30)	30% (9/30)

^***^ *p* < 0.001.

^****^ *p* < 0.0001.

### 3.4 BMI Sub‐Analysis in Healthy Volunteers

Because larger B_0_ fluctuations might occur in participants with higher BMI, we performed a subgroup analysis on three participants with BMI > 30 to examine the robustness of the navigator correction in conditions potentially prone to greater field variations. Figure S5 shows representative standard‐ and navigated‐reconstructed slices, with the corresponding WM/GM CNR measurements across all slices from these three participants. Across three higher‐BMI participants, navigated reconstruction yielded greater mean CNR values (0.80 ± 0.31, 0.44 ± 0.33, and 1.14 ± 0.47) compared to standard reconstruction (0.57 ± 0.32, 0.39 ± 0.27, and 0.83 ± 0.40), respectively, suggesting that navigator‐based correction remains effective even in conditions with potentially larger B_0_ fluctuation.

### 3.5 Comparison Between Pre‐Echo and Post‐Echo Navigator Methods

A sub‐analysis was performed to directly compare the proposed pre‐echo navigator approach with the post‐echo navigator by Beghini et al. [20] in one representative participant (female, 35 years old). Because each navigator approach required an independent scan acquisition, the standard reconstructed images for each approach reflect different acquisitions with inherent scan‐to‐scan variability. It should be noted that the post‐echo navigator method used shorter echo times (TE = {3.8, 10.6, 16.4} ms vs. {9.5, 16.3, 23.1} ms for pre‐echo), which inherently provides a better baseline image quality independent of the navigator correction. Percent improvements in image quality relative to uncorrected standard images were calculated for both methods due to this variation (Table S1). Both methods demonstrated improvements in navigator‐corrected reconstructions compared to standard reconstructions across all metrics (WM SNR, GM SNR, and CNR). While the pre‐echo navigator showed larger percentage improvements compared to baseline (27.4%–86.2% vs. 5.2%–28.2%), this is influenced by the lower baseline quality of the pre‐echo standard images in addition to navigator performance. In absolute terms, both methods achieved comparable navigator‐corrected image quality (CNR = 1.38 post‐echo vs. 1.63 pre‐echo), with the post‐echo acquisition benefiting from superior baseline quality due to shorter TEs. Representative slices demonstrating B_0_ field fluctuations and corresponding reconstructions for both methods are shown in Figure S6, illustrating that both approaches successfully mitigate breathing‐induced artifacts. The comparable absolute image quality achieved by both methods is consistent with the modest visual differences observed in Figure S6.

### 3.6 Association Between Qualitative and Quantitative Metrics in Healthy Volunteers

Spearman's rank correlation analysis demonstrated strong associations between the quantitative metrics and qualitative Likert Scores, as demonstrated in Figure 6. For the standard reconstruction, artifact Likert scores were correlated with GM SNR (*ρ* = 0.778, *p* < 0.001), WM SNR (*ρ* = 0.701, *p* < 0.0001), ghosting (*ρ* = 0.757, *p* < 0.0001), and CNR (*ρ* = 0.521, *p* = 0.003). Standard structural Likert Scores were also significantly correlated with GM SNR (*ρ* = 0.687, *p* < 0.0001), WM SNR (*ρ* = 0.730, *p* < 0.0001), ghosting (*ρ* = −0.549, *p* = 0.002), and CNR (*ρ* = 0.615, *p* = 0.0003). In the navigator‐corrected reconstruction, artifact Likert scores remained correlated with GM SNR (*ρ* = 0.827, *p* < 0.0001), WM SNR (*ρ* = 0.604, *p* < 0.0001), and ghosting (*ρ* = −0.684, *p* < 0.0001), whereas CNR was not significantly associated (*ρ* = 0.236, *p* = 0.210). Finally, navigated structural Likert scores remained correlated with GM SNR (*ρ* = 0.466, *p* = 0.009), WM SNR (*ρ* = 0.534, *p* = 0.002), and CNR (*ρ* = 0.605, *p* = 0.0004), but were no longer correlated with ghosting (*ρ* = −0.129, *p* = 0.497).

**FIGURE 6:**
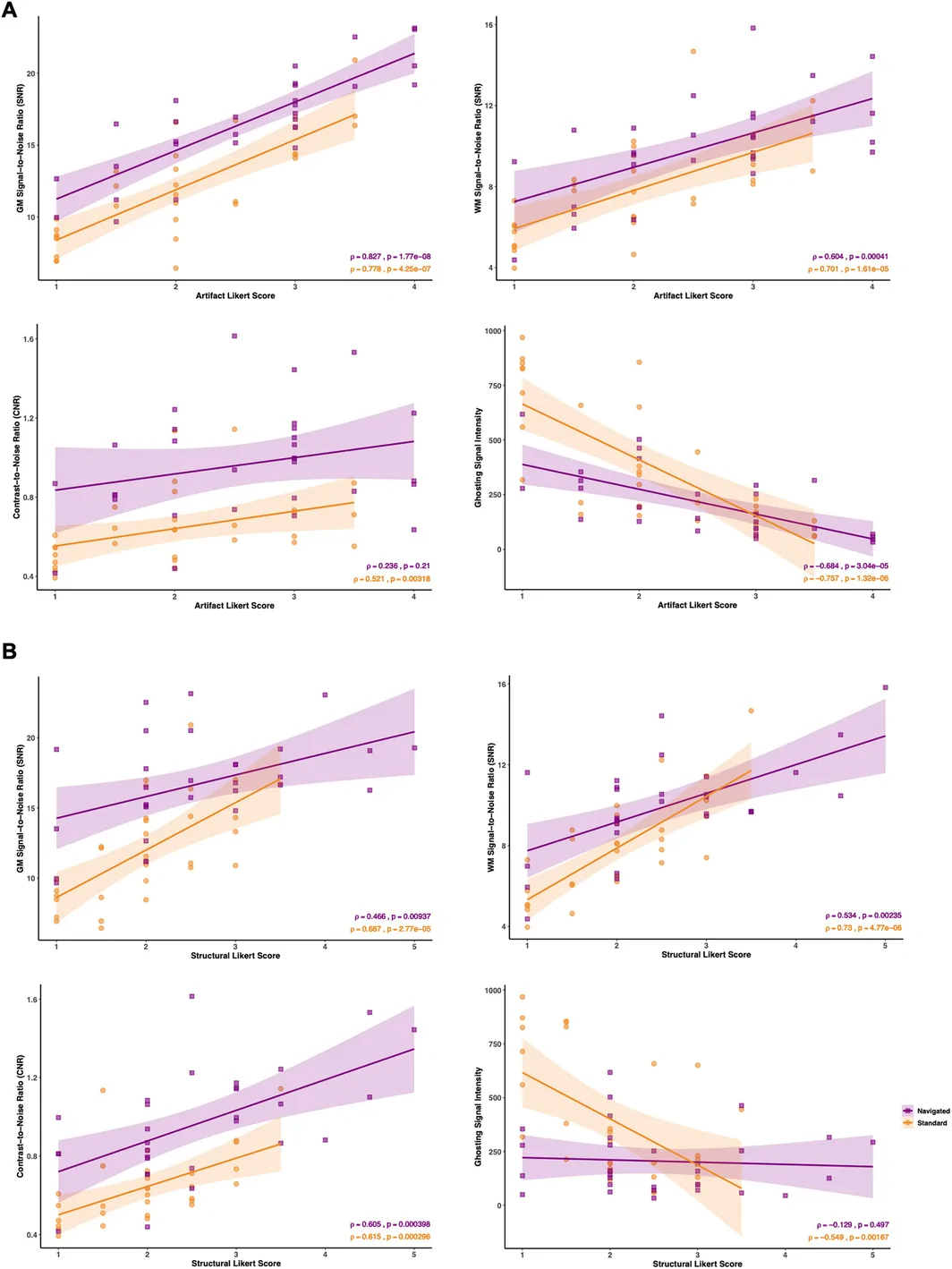
(A) Spearman rank correlations between mean artifact Likert score (between the two raters) and the mean quantitative image quality metrics in all healthy volunteers (*n* = 20) for both the standard and navigator‐corrected reconstructed images. All are significantly correlated except for navigator‐corrected reconstructed CNR. (B) Spearman rank correlations between mean structural Likert score (between the two raters) and the mean quantitative image quality metrics in all healthy volunteers (*n* = 20) for both the standard and navigator‐corrected reconstructed images. All are significantly correlated except for the navigator‐corrected reconstructed ghosting signal intensity.

### 3.7 pwRRMS Qualitative Image Quality Evaluation

Figure 7 illustrates representative upper and lower thoracic spinal cord slices of a pwRRMS reconstructed with the standard and navigator‐correction methods. Across the slices in the lower thoracic spinal cord of the pwMS, the navigator‐correction reconstruction visibly reduced artifact and improved the structural delineation of the spinal cord to CSF boundary, and GM to WM contrast. Lesions also appeared more conspicuous on the navigated images, with better delineation of lesion boundaries. The upper thoracic spinal cord slices showed little qualitative difference between the standard and navigator‐corrected reconstructions; however, the reconstruction method does not appear to produce lower‐quality images.

**FIGURE 7:**
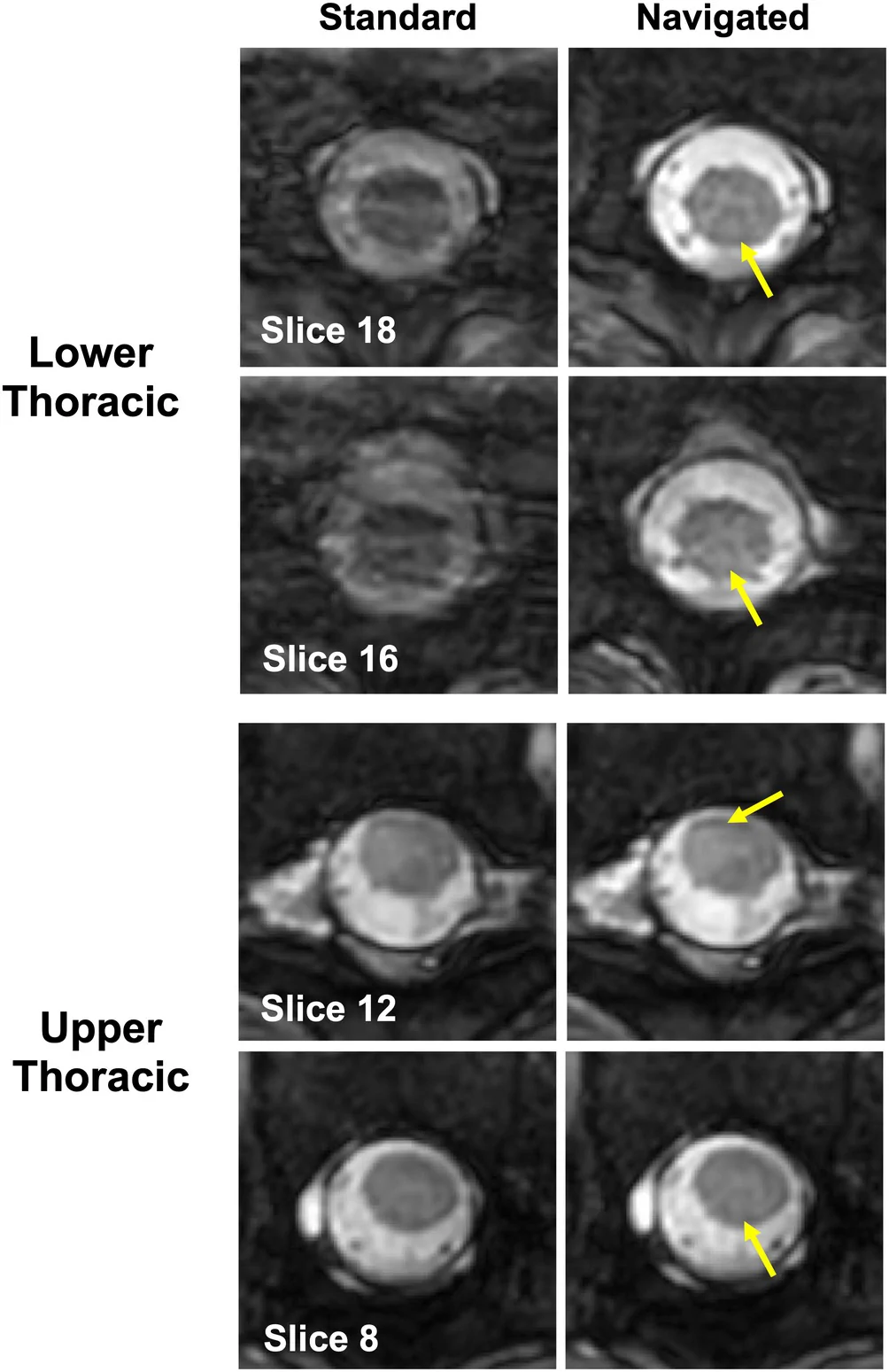
Example ME‐GRE slices in both the upper and lower thoracic spinal cord in a person with relapsing–remitting multiple sclerosis (42‐year‐old female) for both standard (left) and navigator‐corrected (right) reconstruction methods. The yellow arrows highlight areas of lesioned tissue in the cord.

In the three pwRRMS, a blind qualitative assessment using a Likert scale was performed to evaluate artifacts, structural delineation, and diagnostic confidence. In the upper and lower thoracic combined region, the mean Likert score of the standard reconstruction for artifact visibility was 2.25 ± 0.75, while for navigator‐correction reconstruction it was 3.33 ± 0.65. Further, the mean standard reconstruction score for structural delineation was 2.25 ± 045, and the mean navigator‐correction reconstruction score was 2.67 ± 0.65. Finally, the standard reconstruction had a diagnostic confidence score of 2.17 ± 0.58, while the navigator‐correction diagnostic score was 2.83 ± 0.72. Figure 8 demonstrates that these blind qualitative ratings were directionally consistent with our visual observations. Artifact scores improved in both the upper and lower thoracic spinal cord for all three pwRRMS. Structural delineation improved in two of the three pwRRMS, with one of the pwRRMS having the same structural score in both the upper and lower thoracic spinal cord. Finally, diagnostic confidence was higher in five of the six paired images, with only one pwRRMS upper thoracic spinal score remaining consistent. Although statistical comparisons were not performed due to the small sample size (*n* = 3 with both upper and lower thoracic images), the consistent directional improvement across the regions and participants supports the qualitative observations in Figure 7.

**FIGURE 8:**
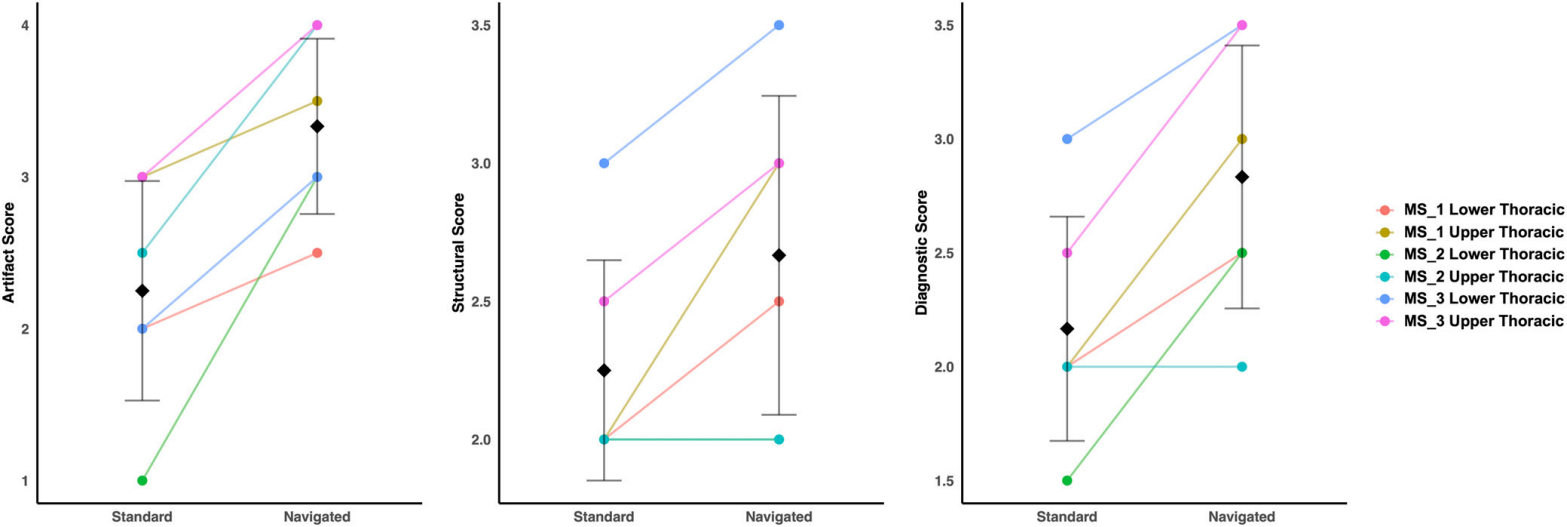
Paired Likert data of the pwRRMS (*n* = 3) in both the upper and lower thoracic spinal cord in the standard‐ and navigator‐corrected reconstructed images. This paired data demonstrates that, across the artifact, structural, and diagnostic Likert scores, image quality improves in the navigated images. Each circle represents the mean Likert score between the two raters for each patient. The black diamond represents the mean Likert score for each of the three patients for both the upper and lower thoracic images, with the standard deviation.

## 4 Discussion

Building on the prior work that placed the navigator readout after the series of echoes [20], this study evaluated whether positioning the navigator prior to the first ME‐GRE echo improves quantitative and qualitative image quality of the thoracic spinal cord. The proposed navigator‐corrected reconstruction significantly increased GM and WM SNR across the upper, lower, and combined thoracic regions, with parallel increases in CNR between GM and WM and significant reductions in posterior ghosting. Importantly, these improvements were observed in both the mean and median metrics, indicating that the navigator was beneficial across participants and results were not driven by outliers. Qualitative assessment of healthy volunteers demonstrated significantly improved artifact reduction and structural delineation with the correction, particularly in the lower thoracic spinal cord. Correlation analysis further revealed that both artifact severity and structural quality were influenced by ghosting in the standard reconstruction, whereas once corrected by the navigator, structural delineation was no longer associated with ghosting and primarily related to the intrinsic signal measures of CNR and SNR. In pwRRMS, navigator‐corrected images demonstrated consistent directional improvements in artifact mitigation, structural delineation, and diagnostic confidence, with lesions appearing more conspicuous on the navigated images. Together, the findings of this study demonstrate that the navigator correction overall improves thoracic spinal cord ME‐GRE imaging by reducing respiratory‐related phase artifacts and recovering signal stability.

The proposed navigator pipeline places the navigator prior to the first echo, which provides several technical advantages compared to the previous study, in which the navigator readout was performed following the echoes [20]. In the study by Behigini et al. in which the navigator was placed after the last echo, the measured phase reflects both dynamic B_0_ fluctuations and accumulated navigator TE‐dependent phase evolution. To resolve this phase ambiguity, spatial interval selection, spinal cord localization, and temporal phase unwrapping using respiratory bellows data were used [20]. In contrast, by acquiring the navigator readout prior to the *k*‐space traversal and at a short TE, a more direct estimate of the instantaneous global frequency offset is obtained, which influences the subsequent signal evolution. Because this estimate is obtained before substantial spatially varying phase dispersion develops, a slice‐wise correction derived from the center *k*‐space sample was sufficient. The initial assumption was that the spinal cord would have to be centered in the FOV. However, this study demonstrated that navigator correction performance was robust to positioning variability as there were no significant correlations found between centering accuracy and the change in image‐quality metrics. This robustness reflects the spatially smooth nature of respiratory‐induced B_0_ fluctuations and shows that they have relative slice‐wise uniformity close to the spinal cord. This finding aligns with previous studies [7, 18, 24], and makes physical sense given the small diameter of the spinal cord is negligible compared to the scale of respiratory‐induced B_0_ changes, so these effects are expected to be mostly uniform within each slice [7, 24]. Under these conditions, slice‐wise correction is theoretically justified. Eliminating projection‐based region selection and phase unwrapping reduces sensitivity to segmentation inaccuracies, anatomical variability, and low‐SNR phase instability, while also simplifying the reconstruction pipeline and mitigating unwrapping failures, which occur in regions with large susceptibility‐induced fluctuations [20]. From a clinical implementation perspective, the absence of external hardware requirements and the ability to perform real‐time reconstruction without specialized post‐processing (i.e., phase unwrapping) make this approach more practical for adoption. While the short TE of the navigator (5.15 ms) acquisition minimizes the need for temporal phase unwrapping, this assumption may not hold in all cases. Severe or heterogeneous B_0_ fluctuations could result in phase wrapping, potentially compromising the accuracy of the frequency offset estimate. Future work should evaluate conditions under which phase unwrapping becomes necessary.

This study demonstrated that navigator‐correction reconstruction resulted in a significant increase in both GM and WM SNR, accompanied by a substantial reduction in posterior ghosting. The navigator pipeline does not alter the intrinsic tissue signal, suggesting that improvements reflect improved temporal phase stability. In ME‐GRE acquisitions, respiratory‐induced B_0_ fluctuations introduce time‐varying frequency offsets that can modify the phase evolution during and between echo acquisitions [10, 25]. Because phase accrual scales linearly with echo time, dynamic field shifts produce echo‐dependent phase errors that vary across TR. This can lead to *k*‐space phase errors, manifesting as ghosting and signal‐intensity changes [8, 24], thereby reducing SNR. By estimating and correcting the instantaneous frequency offset before echo acquisition, the navigator can reduce temporal phase variation and mitigate signal loss. It should also be noted that both the mean and median image quality metrics consistently improved in the navigator‐correction reconstruction, suggesting that this improvement is not driven by outliers.

In the sub‐analysis, both the proposed navigator (i.e., pre‐echo series) and the navigator proposed by Beghini et al. (i.e., post‐echo series) [20] demonstrated improvements in image quality even though they differ in practical considerations and reconstruction pipelines. While the pre‐echo navigator demonstrated larger percentage improvements in image quality metrics, this reflects its lower baseline image quality rather than solely navigator performance. The post‐echo navigator acquisition achieved comparable absolute image quality after correction, benefiting from superior baseline quality due to shorter TEs. In this single participant sub‐analysis, both methods demonstrated navigator‐based improvements in their respective acquisitions. The choice between them may depend on sequence design constraints, computational efficiency, and clinical workflow considerations. The pre‐echo approach offers the advantage of eliminating external hardware requirements, while the post‐echo approach may be more readily integrated into existing ME‐GRE sequences. However, direct comparison in this study is limited by differing echo times between methods, independent acquisitions where respiratory induced artifacts can vary between scans, and sample size of one. Future work comparing both methods across larger cohorts would help clarify which approach is optimal for different imaging scenarios.

Correlation analysis between quantitative and qualitative image quality metrics in healthy volunteers revealed a shift in the factors underlying perceived image quality following navigator‐correction reconstruction in the ME‐GRE images. In the standard reconstruction, both artifact severity and structural delineation were significantly associated with ghosting, demonstrating that the respiratory‐induced phase fluctuations directly contributed to degradation of structural detail. Once corrected, ghosting was no longer significantly correlated with structural quality; however, structural delineation was associated with CNR and SNR, image‐quality metrics associated with signal. These findings suggest that the proposed navigator mitigates structural artifact degradation, allowing the quality of navigated images to be driven by signal rather than artifacts. In spinal cord imaging, this ghosting can obscure subtle anatomical boundaries and details and bias quantitative assessments [18].

Validation of this navigator‐correction pipeline was performed in the thoracic spinal cord, motivated by both physics and clinical considerations. The thoracic spinal cord has close proximity to large susceptibility interfaces at the lung borders, approximately at the vertebral levels C7‐T1 superiorly and T8–T11 inferiorly, where respiratory‐induced B_0_ perturbations are largest and most heterogeneous [20]. In our study, we chose a region where B_0_ fluctuations would be largest (vertebral levels T9–T12, or lower thoracic), and a region where fluctuations are less pronounced (vertebral levels T4–T8, or upper thoracic). Demonstrating robust correction under both of these conditions suggests that the proposed navigator could also be translated to spinal cord regions with less pronounced susceptibility variation, such as the cervical and lumbosacral spinal cord. Clinically, the thoracic spinal cord is relevant for the diagnosis, prognosis, and treatment planning of diseases such as MS [26, 27], trauma [28], and degeneration [29], yet imaging these regions is technically demanding. Any improvements in artifact mitigation and signal stability in the thoracic spinal cord may substantially enhance the reliability and reproducibility of spinal cord imaging, and thereby the diagnostic reliability and lesion conspicuity.

Respiratory B_0_ fluctuations can vary between individuals (e.g., due to height and weight, respiration rate) and across imaging sessions [7, 24, 30], potentially introducing variability in both qualitative interpretation and quantitative measurements in multi‐echo acquisitions. By mitigating respiratory phase variability during acquisition, the navigator‐correction may reduce inter‐session variability and improve reproducibility, which is ultimately important for longitudinal disease monitoring. However, it should be noted that this study was performed in a cohort with an average BMI of 25.1 kg/m^2^ and while three participants had a BMI higher than 30, the results from this cohort may not necessarily be representative of patient populations who have higher BMI, as it has been shown that field fluctuations tend to increase with higher BMI [31]. Our sub‐analysis demonstrated that the navigator‐corrected images maintained superior CNR on average across the image volume in these participants; however, we acknowledge that validation in a larger and more diverse cohort would strengthen this conclusion. Furthermore, improved SNR and CNR in the navigator‐correction may reduce the number of averages needed, allowing for shorter acquisition times or higher spatial resolution within clinically feasible scan times, but future studies are needed to validate this. Reduction of ghosting could also improve the robustness of automated segmentations and post‐processing pipelines.

Since this study focused on an ME‐GRE acquisition, there are possible extensions beyond improved image quality in ME‐GRE that could be achieved by using the navigator. Echo‐dependent phase accrual arising from dynamic B_0_ fluctuations can introduce signal changes and ghosting, and bias quantitative measurements, including R2* and susceptibility measures [25]. Stabilizing the instantaneous frequency offset prior to an echo acquisition may reduce echo‐dependent variability and improve the robustness and reproducibility of multi‐echo quantitative metrics, which are important for longitudinal studies. Although we did not implement this navigator for susceptibility or R2* measurements, it would be of interest to evaluate whether this proposed navigator can also enhance the reliability of these quantitative metrics along the spinal cord.

There are several limitations to consider in this study. First, this current study has a limited sample size of both healthy participants and pwRRMS. To further investigate and understand the implications of the anatomical ME‐GRE navigator correction in a clinical population (e.g., MS), a larger patient cohort needs to be recruited, and image quality evaluated. Future studies with a larger MS cohort are needed to establish whether the navigator improves lesion detection or diagnostic confidence. Additionally, to quantify the reliability of the navigated ME‐GRE, a longitudinal study in healthy participants should be performed to ensure that image quality improvements are reproducible across sessions. Furthermore, while image quality mostly improved in the navigator‐corrected reconstructed images, some standard reconstructions had better image quality, as demonstrated by some individual paired lines showing the opposite trend to the majority (shown in both Figures 3 and 4). This could be because the approach assumes the navigator pixel represents the cord region being imaged. While we tried to center the spinal cord in the middle of the FOV, significant participant movement prior to acquisition or a significantly curved spinal cord might make this not hold true for all acquisitions. A future study should be performed in which the navigator pipeline is modified to account for phase fluctuations across the entire spinal cord by incorporating automatic cord segmentation in SCT to determine if this improves the robustness of the navigator to anatomical variability.

It should also be noted that ghosting artifacts were only quantified within an ROI centered posteriorly to the spinal cord. Ghosting could be more severe to the periphery of the FOV. A more comprehensive spatial analysis of ghosting across the full FOV could be performed in future studies to fully characterize the respiratory‐induced motion artifacts. Additionally, the 1D navigator used in this study acquires spatial information exclusively in the readout direction, limiting the correction to that dimension. While respiratory motion is primarily through‐plane [7, 18, 24], this current approach does not detect field variations in other directions. The use of a 2D or 3D navigator could provide a more comprehensive correction; however, at the cost of increased acquisition complexity and time. Finally, while the navigator mitigates respiratory‐induced through‐plane B_0_ fluctuations, dynamic susceptibility gradients may introduce residual temporal signal dephasing that cannot be fully addressed. Prospective real‐time shimming [15, 16, 17, 19, 32], along with the navigator, may address these effects in future studies.

## 5 Conclusion

The findings from this study demonstrate that placing a navigator readout before the first echo and at a short TE allows direct estimation of the breathing‐induced B_0_ field fluctuations in the thoracic spinal cord, enabling efficient slice‐wise phase correction without temporal phase unwrapping using respiratory bellows. This approach mitigates respiratory‐related phase fluctuations in the thoracic spinal cord, suppressing ghosting artifacts, increasing SNR and CNR, and shifting the determinants of image quality in anatomical ME‐GRE acquisitions from artifact‐dominated to relying on the intrinsic signal of tissue. The proposed navigator‐corrected pipeline is a streamlined approach that has implications for improving the diagnostic value of ME‐GRE imaging, increasing the reliability of quantitative analyses, and enhancing longitudinal reproducibility in spinal cord imaging.

## Funding

This work was supported by the National Institutes of Health, K01EB030039, R03TR004434; Canada Research Chair in Quantitative Magnetic Resonance Imaging, CRC‐2020‐00179; Natural Sciences and Engineering Research Council of Canada, PDF, RGPIN‐2019‐07244; National Multiple Sclerosis Society, JF‐2306‐41540; Santé, 322736, 324636; Canada First Research Excellence Fund; Canadian Institutes of Health Research, PJT‐190258, PJT‐203803; Canada Foundation for Innovation, 32454, 34824; Quebec BioImaging Network, 35450, 5886; Courtois NeuroMod project.

## Conflicts of Interest

Ryan K. Robison is an employee of Philips North America LLC. Guillaume Gilbert is an employee of Philips Healthcare Canada.

## Supporting information


**Figure S1:** Histograms of slice‐wise displacement of the spinal cord mask centroid from the center of the image field‐of‐view in the right–left (R‐L) direction for the lower (*n* = 23, orange) and upper (*n* = 13, blue) thoracic spinal cord across all participants. The middle‐dashed line indicates zero displacement (perfect centering). The mean spinal cord mask width in the R‐L direction across all scans was 31 voxels, indicating that the overall displacement was small relative to the cord width (shown with the dashed lines at −15.5 and 15.5 displacement).
**Figure S2:** Spearman rank correlations between mean quantitative image quality metric differences (navigated minus standard for CNR, GM SNR, WM SNR, and ghosting) and absolute mean displacement in the right–left direction from the center of the image FOV for both the upper thoracic (*n* = 13) and lower thoracic (*n* = 23) region. No significant correlations were found among the quantitative image metrics, suggesting that correction performance was not meaningfully sensitive to the spinal cord position within the FOV.
**Figure S3:** (A) Representative sagittal Dixon GRE water image of one healthy participant (35‐year‐old female), with ROIs highlighted in orange (T9), green (T11–T12), and purple (L1). (B) Representative dynamic Dixon B_0_ images (dynamics 3, 49, and 96), demonstrating change in B_0_ throughout respiration. (C) The B_0_ time series of the three ROIs from the Dixon B_0_ maps (centered around 0 Hz), which align with the respiratory trace from the bellows (dashed black line). DB_0_ offsets as large as roughly 40 Hz were measured.
**Figure S4:** Example ME‐GRE images in the upper and lower thoracic spinal cord in three healthy participants, with the standard‐reconstruction (no navigator) and navigator‐corrected reconstruction displayed. Pairwise differences between the standard and navigated reconstructed images were computed after normalization (dividing each image by the maximum intensity value in that image).
**Figure S5:** Representative axial ME‐GRE images in the lower thoracic spinal cord from three participants with BMI > 30, comparing standard reconstruction (left) and navigated reconstruction (right) images. Across the representative slices, the navigator demonstrated improved image quality compared to standard reconstruction. White/gray matter CNR measurements across all slices for each participant for the standard‐ (orange) and navigated‐ (purple) reconstructed images are also shown, with the mean CNR (with standard deviation) across slices shown for the standard‐ (black circle) and navigated‐ (black square) reconstructed images. This demonstrates that overall, the navigator‐corrected images have higher CNR despite the potentially greater B_0_ fluctuations of the higher BMI participants. Missing CNR data points are due to the inability to delineate and segment the gray matter on that slice.
**Figure S6:** (A) Navigator placed prior to the echo train (“Pre‐Echo Navigator”). Representative slices of the ME‐GRE images in the lower thoracic spinal cord of one healthy participant (35‐year‐old female), with the standard‐reconstruction (no navigator) and navigator corrected reconstruction displayed. Measured B_0_ offset values from the ME‐GRE sequence during free breathing were roughly −20 to 10 Hz (right column). (B) Navigator placed after the echo train based on work performed by Beghini et al. (“Post‐Echo Navigator”). Note that phase unwrapping was performed. Representative slices of the ME‐GRE images in the lower thoracic spinal cord of one healthy participant (35‐year‐old female), with the standard‐reconstruction (no navigator) and navigator‐corrected reconstruction displayed. Measured B_0_ offset values from the ME‐GRE sequence during free breathing were roughly −10 to 15 Hz (right column).
**Table S1:** Comparison of image quality metrics between pre‐echo and post‐echo navigator methods in one participant (35‐year‐old female). Standard and navigated SNR and CNR values in the table are the mean (±SD) of all slices in the image volume. Percent difference is calculated relative to the standard reconstruction image metric.

## Data Availability

All example scripts to quantify image quality, along with documentation, are available online (https://github.com/sct-pipeline/gre-1dnav-analysis/tree/v1.0). All data used in this study are available for download online (https://openneuro.org/datasets/ds006347).
