# Supplementary material for: 1D Pre‐Acquisition Navigator Correcting Respiratory‐Induced Field Fluctuations in Multi‐Echo Gradient‐Echo Imaging of the Thoracic Spinal Cord
Source: Magn Reson Med. 2026 Jul 24;96(6):2660–74. doi: 10.1002/mrm.70528 (PMC13619845; doi:10.1002/mrm.70528)
Supplement: Supplementary file 1 [file MRM-96-2660-s001.pdf]

Supplementary Materials

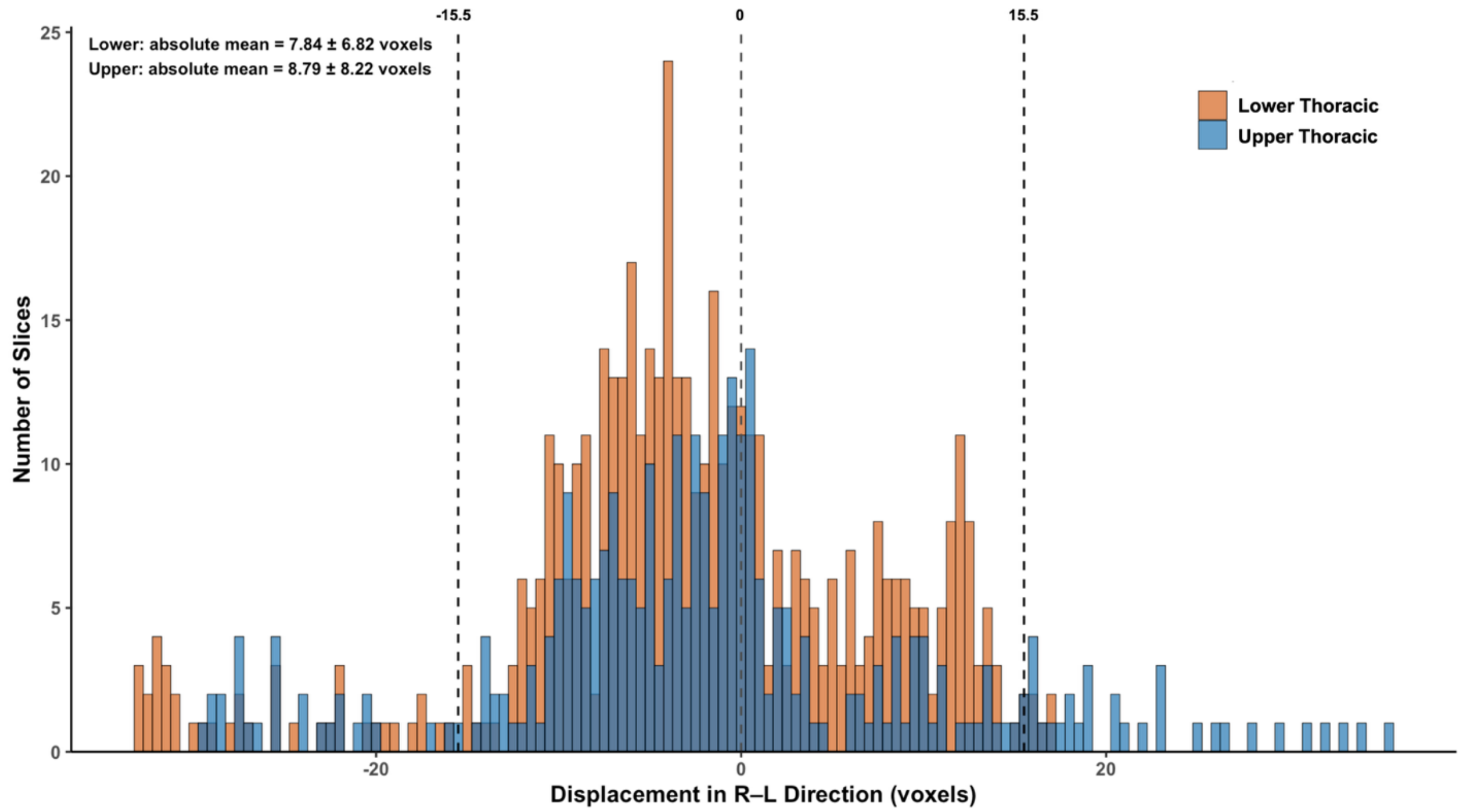

Figure S1: Histograms of slice-wise displacement of the spinal cord mask centroid from the center of the image field-of-view in the right-left (R-L) direction for the lower (n = 23, orange) and upper (n = 13, blue) thoracic spinal cord across all participants. The middle-dashed line indicates zero displacement (perfect centering). The mean spinal cord mask width in the R-L direction across all scans was 31 voxels, indicating that the overall displacement was small relative to the cord width (shown with the dashed lines at -15.5 and 15.5 displacement).

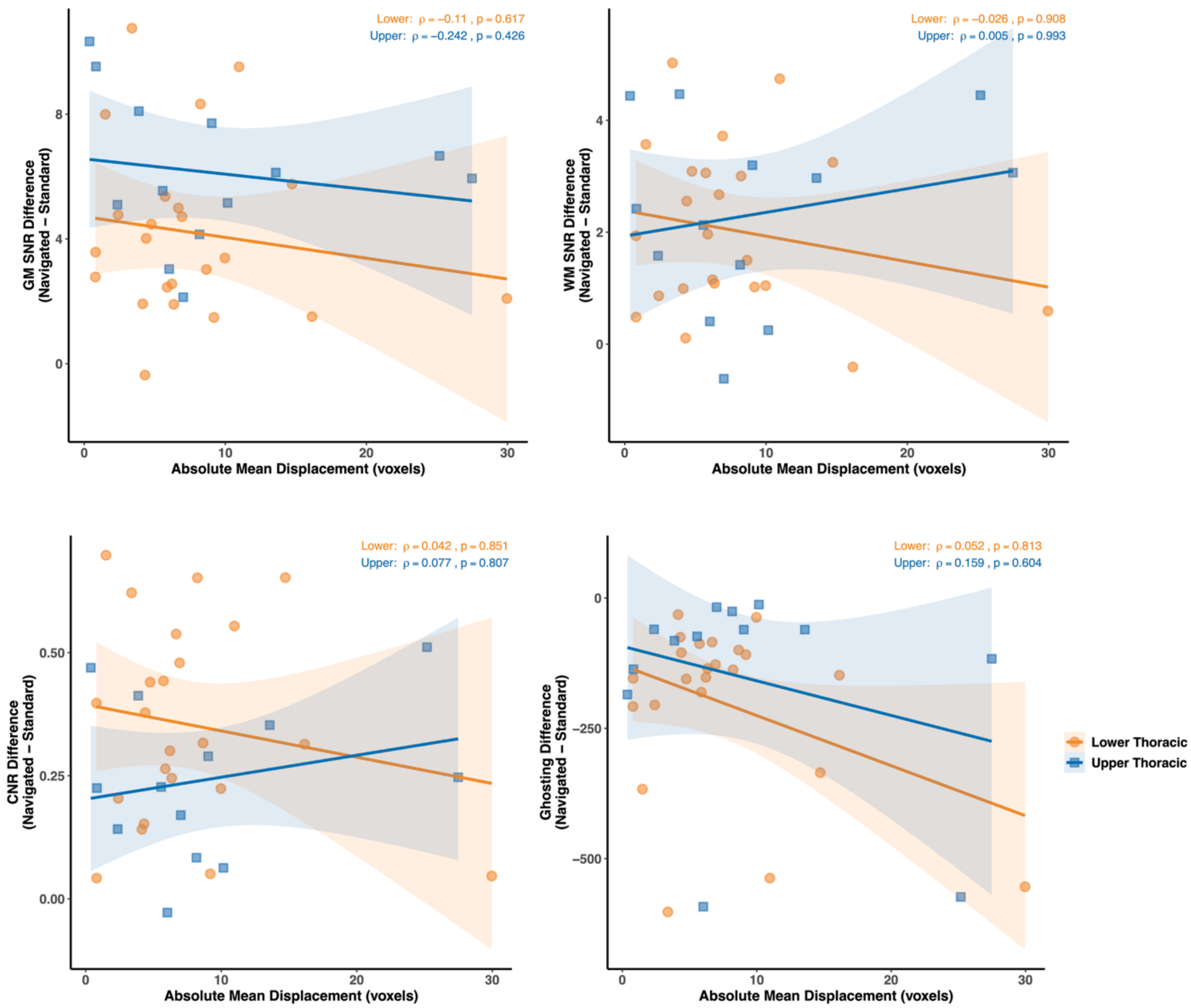

Figure S2: Spearman rank correlations between mean quantitative image quality metric differences (navigated minus standard for CNR, GM SNR, WM SNR, and ghosting) and absolute mean displacement in the right-left direction from the center of the image FOV for both the upper thoracic (n = 13) and lower thoracic (n = 23) region. No significant correlations were found among the quantitative image metrics, suggesting that correction performance was not meaningfully sensitive to the spinal cord position within the FOV.

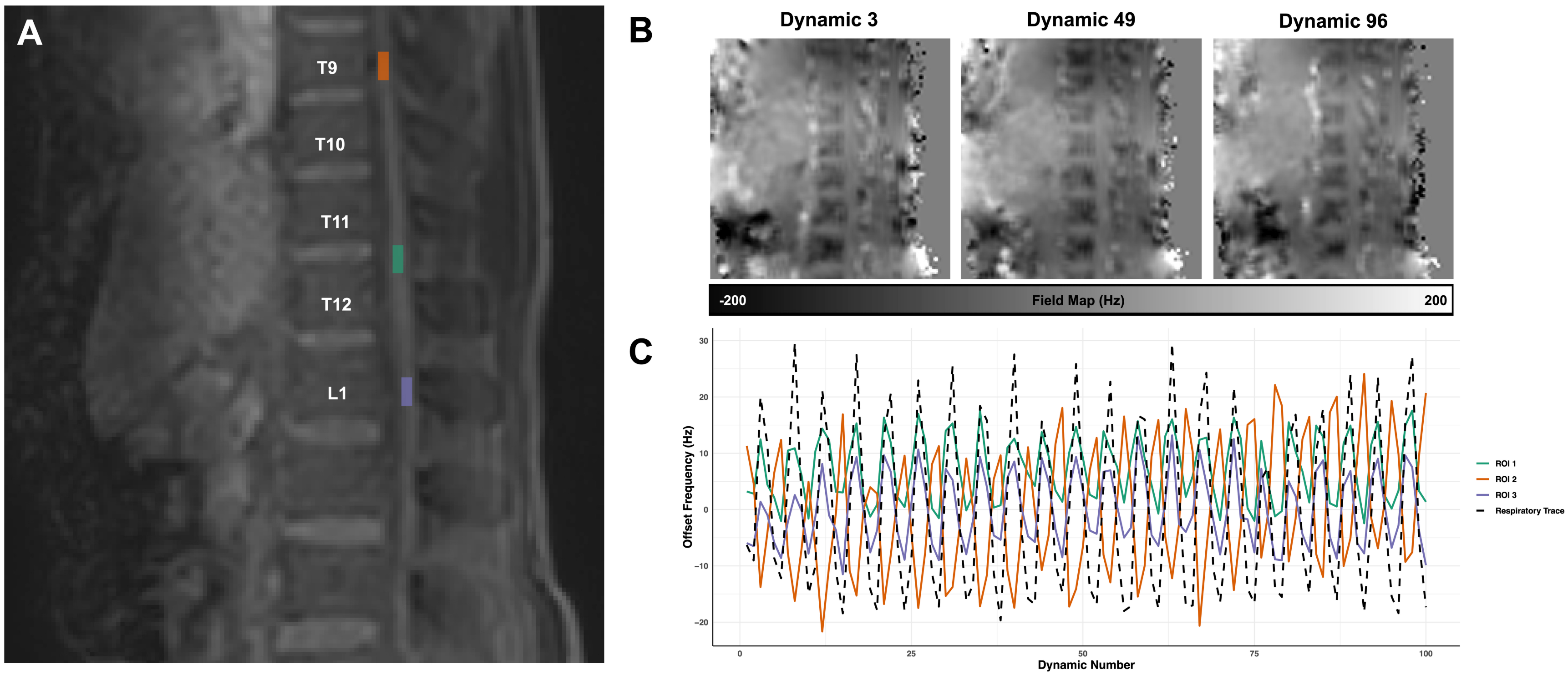

Figure S3: **A.** Representative sagittal Dixon GRE water image of one healthy participant (35-year-old female), with ROIs highlighted in orange (T9), green (T11-T12), and purple (L1). **B.** Representative dynamic Dixon B_0 images (dynamics 3, 49, and 96), demonstrating change in B_0 throughout respiration. **C.** The B_0 time series of the three ROIs from the Dixon B_0 maps (centered around 0 Hz), which align with the respiratory trace from the bellows (dashed black line). ΔB_0 offsets as large as roughly 40 Hz were measured.

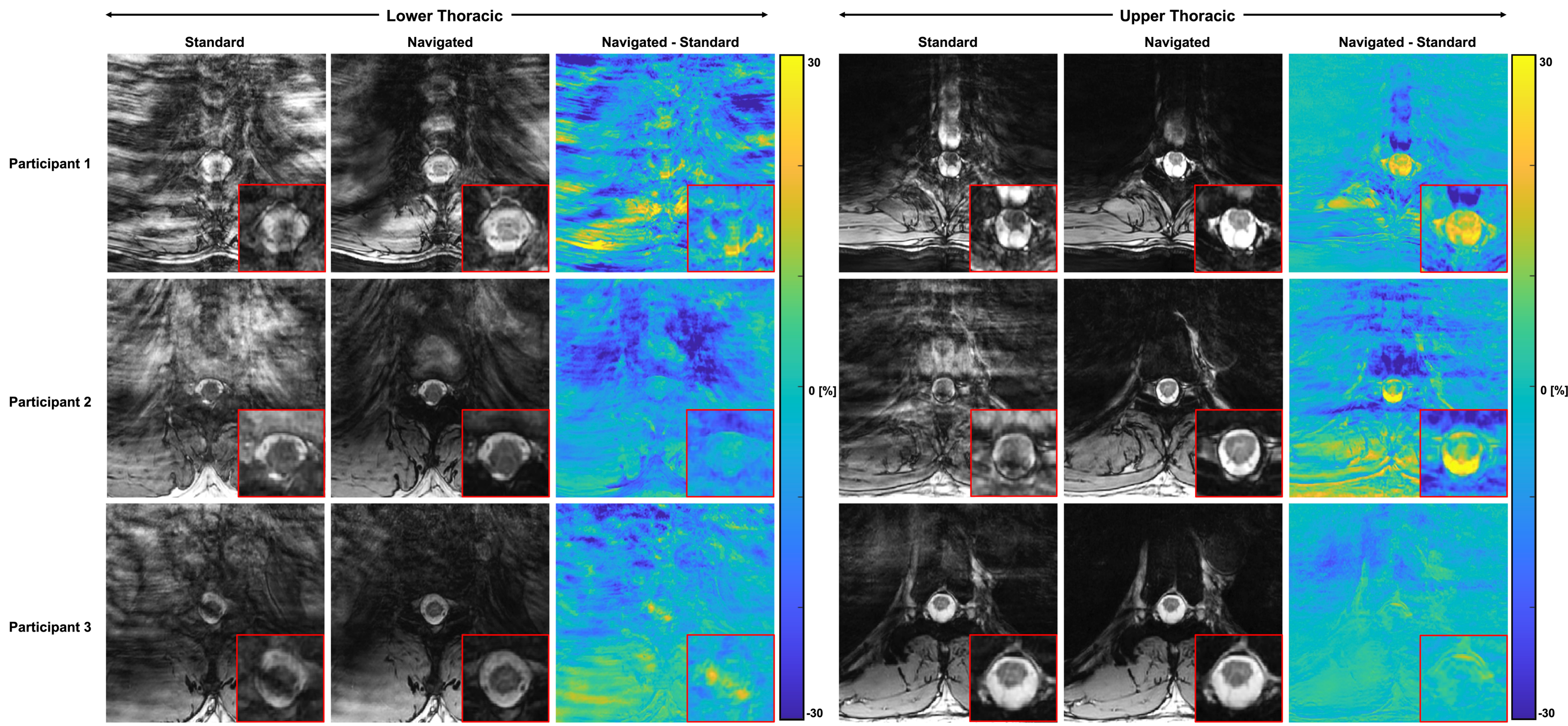

Figure S4: Example ME-GRE images in the upper and lower thoracic spinal cord in three healthy participants, with the standard-reconstruction (no navigator) and navigator-corrected reconstruction displayed. Pairwise differences between the standard and navigated reconstructed images were computed after normalization (dividing each image by the maximum intensity value in that image).

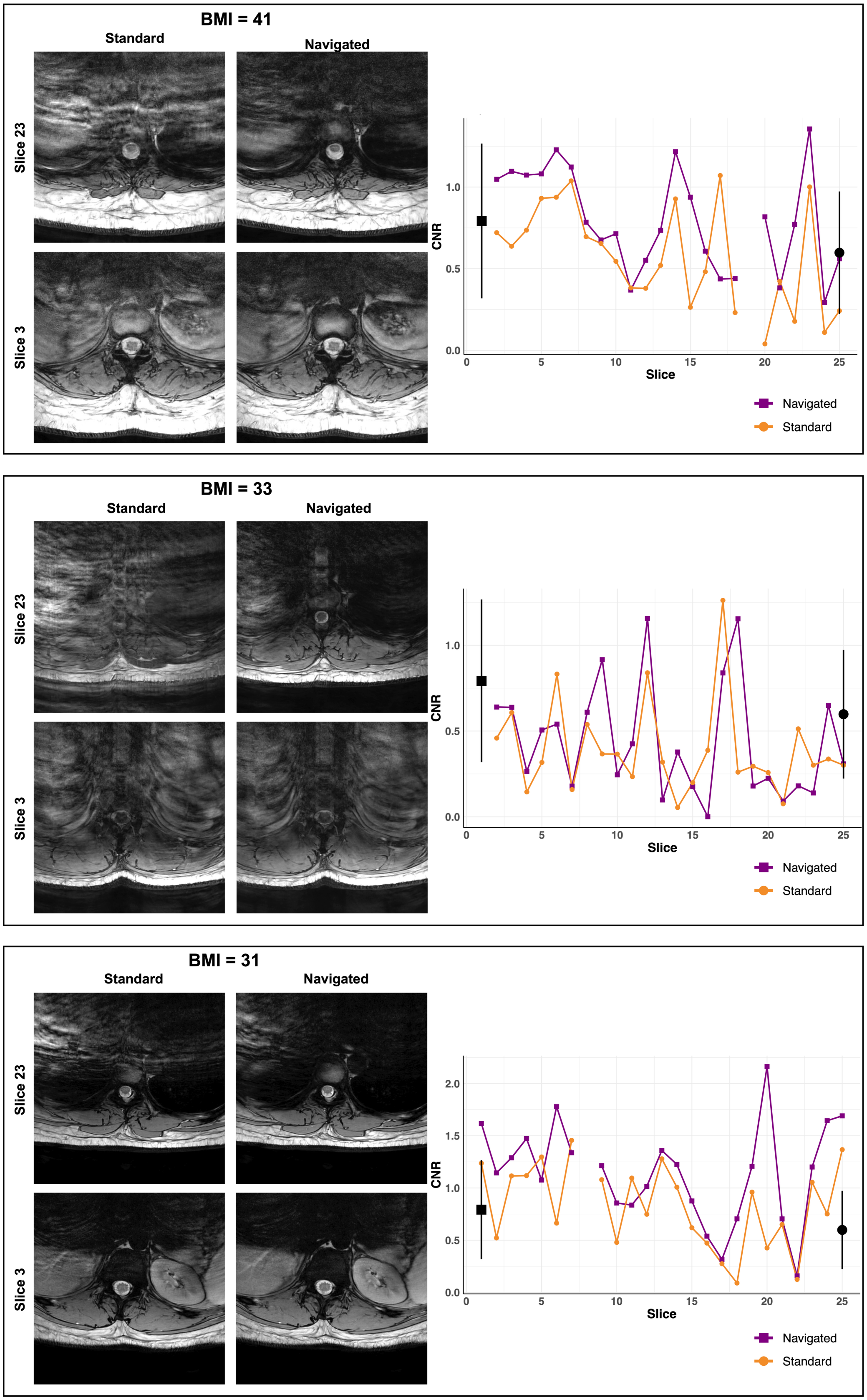

BMI = 41
Standard
Navigated
Slice 23
Slice 3
CNR
Slice
Navigated
Standard
BMI = 33
Standard
Navigated
Slice 23
Slice 3
CNR
Slice
Navigated
Standard
BMI = 31
Standard
Navigated
Slice 23
Slice 3
CNR
Slice
Navigated
Standard

Figure S5: Representative axial ME-GRE images in the lower thoracic spinal cord from three participants with BMI >30, comparing standard reconstruction (left) and navigated reconstruction (right) images. Across the representative slices, the navigator demonstrated improved image quality compared to standard reconstruction. White/gray matter CNR measurements across all slices for each participant for the standard- (orange) and navigated- (purple) reconstructed images are also shown, with the mean CNR (with standard deviation) across slices shown for the standard- (black circle) and navigated- (black square) reconstructed images. This demonstrates that overall, the navigator-corrected images have higher CNR despite the potentially greater B_0 fluctuations of the higher BMI participants. Missing CNR data points are due to the inability to delineate and segment the gray matter on that slice.

Table S1: Comparison of image quality metrics between pre-echo and post-echo navigator methods in one participant (35-year-old female). Standard and navigated SNR and CNR values in the table are the mean (±SD) of all slices in the image volume. Percent difference is calculated relative to the standard reconstruction image metric.

	SNR GM			SNR WM			CNR		
	**Standard**	**Nav**	**% Diff**	**Standard**	**Nav**	**% Diff**	**Standard**	**Nav**	**% Diff**
**Pre-echo Navigator**	17.8 ± 8	22.8 ± 5	27.4%	9.4 ± 3	13.4 ± 4	43%	0.88 ± 0.25	1.63 ± 0.46	86.2%
**Post-echo Navigator**	23.2 ± 7	24.5 ± 8	5.2%	16.0 ± 6	17.0 ± 6	6.6%	1.1 ± 0.56	1.38 ± 0.49	28.2%

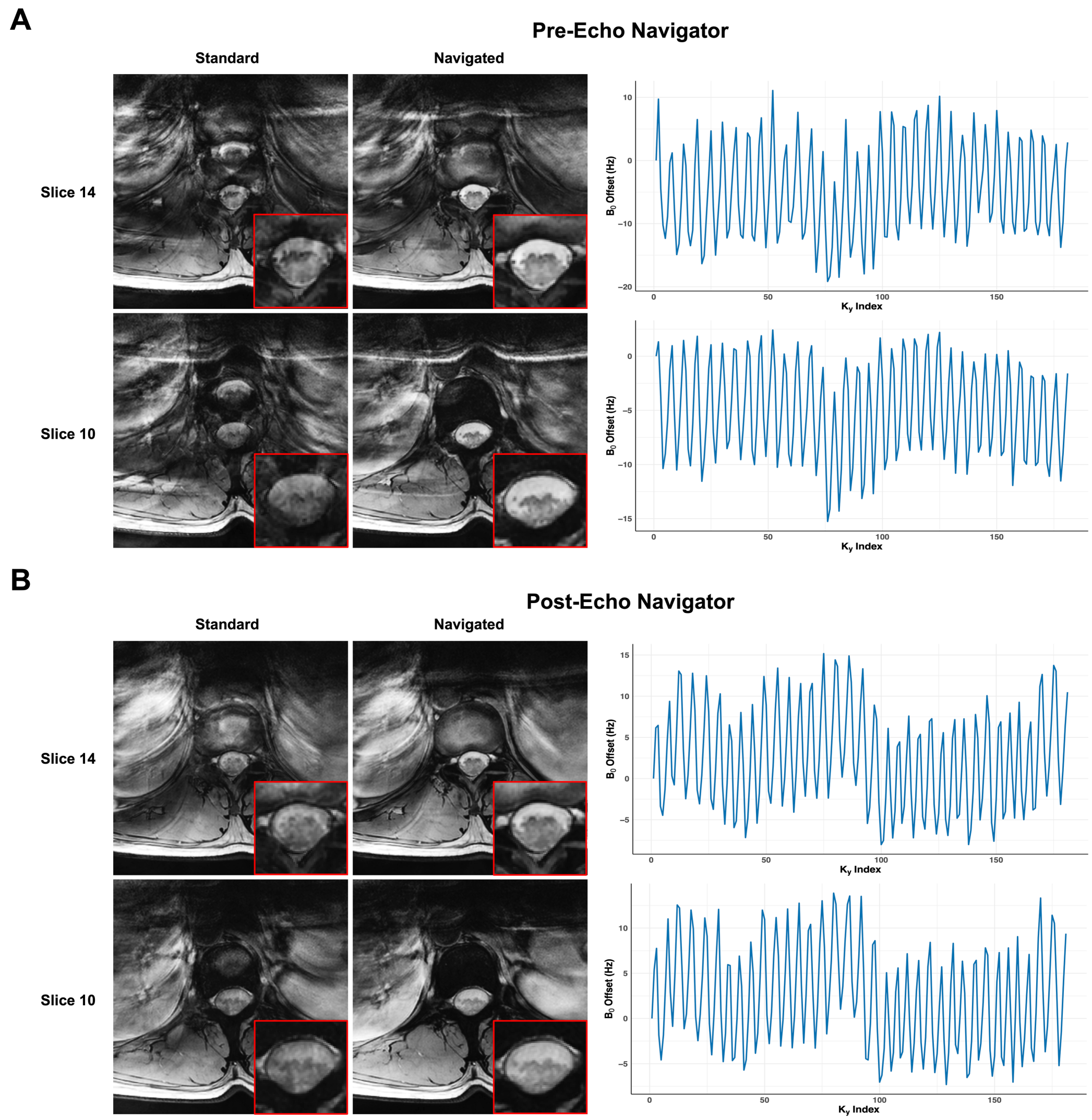

Figure S6: A. Navigator placed prior to the echo train ("Pre-Echo Navigator"). Representative slices of the ME-GRE images in the lower thoracic spinal cord of one healthy participant (35-year-old female), with the standard-reconstruction (no navigator) and navigator-corrected reconstruction displayed. Measured B_0 offset values from the ME-GRE sequence during free breathing were roughly -20-10 Hz (right column). **B.** Navigator placed after the echo train based on work performed by Beghini et al. ("Post-Echo Navigator"). Note that phase unwrapping was performed. Representative slices of the ME-GRE images in the lower thoracic spinal cord of one healthy participant (35-year-old female), with the standard-reconstruction (no navigator) and navigator-corrected reconstruction displayed. Measured B_0 offset values from the ME-GRE sequence during free breathing were roughly -10-15 Hz (right column).
